# All-Cause Maternal Mortality in the US Before vs During the COVID-19 Pandemic

**DOI:** 10.1001/jamanetworkopen.2022.19133

**Published:** 2022-06-28

**Authors:** Marie E. Thoma, Eugene R. Declercq

**Affiliations:** 1 Department of Family Science, School of Public Health, University of Maryland, College Park; 2 Department of Community Health Sciences, School of Public Health, Boston University, Boston, Massachusetts

## Abstract

This cross-sectional study analyzes the factors associated with deaths during and after pregnancy among Black, Hispanic, and White women.

## Introduction

The National Center for Health Statistics (NCHS) reported an 18.4% increase in US maternal mortality (ie, death during pregnancy or within 42 days of pregnancy) between 2019 and 2020. The relative increase was 44.4% among Hispanic, 25.7% among non-Hispanic Black, and 6.1% among non-Hispanic White women.^1^ Given a 16.8% increase in overall US mortality in 2020, largely attributed to the COVID-19 pandemic,^2^ we examined the pandemic’s role in 2020 maternal death rates.

## Methods

This study was exempt from review under the US Department of Health and Human Services regulation for secondary data analysis; consent was not required for vital records. We followed the STROBE reporting guideline.

We used deidentified NCHS mortality and natality files from 2018 to 2020. We limited analyses to maternal deaths (*International Statistical Classification of Diseases and Related Health Problems, Tenth Revision* [*ICD-10*] codes A34, O00-O95, and O98-O99) based on underlying cause of death in accordance with NCHS guidelines.^3^ None of these *ICD-10* codes exclusively identify COVID-19 (*ICD-10* U07.1) as the cause even when COVID-19 was a factor in maternal death (eMethods in the Supplement). Therefore, COVID-19 was ascertained as a secondary cause from the multiple causes-of-death section, consistent with other reports on excess mortality from COVID-19.^4^

Deaths were stratified by month, and year of death was stratified into before (2018, 2019, and January-March [quarter 1] 2020) or during (April-December [quarters 2-4] 2020) the pandemic. Maternal mortality rates and percentages with a secondary COVID-19 code were compared by timing, race and ethnicity, and underlying cause (which were all included in NCHS data). Differences were assessed using a *z* test of proportions. Two-sided *P* = .05 indicated statistical significance.

## Results

A total of 1588 maternal deaths (18.8 per 100 000 live births) occurred before the pandemic vs 684 deaths (25.1 per 100 000 live births) during the pandemic, a relative increase of 33.3% (Table). Late maternal mortality increased by 41%. Absolute and relative changes were highest for Hispanic (8.9 per 100 000 live births and 74.2%, respectively) and non-Hispanic Black (16.8 per 100 000 live births and 40.2%) vs non-Hispanic White (2.9 per 100 000 live births and 17.2%) women (Figure). A secondary code for COVID-19 was listed in 14.9% (102 of 684) of maternal deaths in quarters 2 to 4, with 0% in quarter 1 of 2020. This percentage was highest among Hispanic women (32.1%), followed by non-Hispanic Black (12.9%) and non-Hispanic White (7.3%) women.

**Table.** Cause-Specific Maternal Mortality Rates Before vs During the COVID-19 Pandemic and Percentage of Secondary COVID-19 Cause Codes

Obstetric cause-of-death category (*ICD-10* O-codes)^c^	Prepandemic^a^		During pandemic^b^		Relative change in rates, %^e^	Absolute change in rates per 100 000 live births	*P* value^f^	COVID-19 (*ICD-10* U07.1) listed as a multiple cause in quarters 2-4 2020, No. (%)^g^
	No. (rate per 100 000 live births)^d^	95% CI	No. (rate per 100 000 live births)^d^	95% CI				
Total coded to maternal causes during pregnancy or within 42 d after pregnancy (A34, O00-O95, O98-O99)	1588 (18.8)	17.9-19.8	684 (25.1)	23.2-27.0	33.3	6.3	<.001	102 (14.9)
Obstetric death of unspecified cause (O95)	57 (0.7)	0.5-0.9	12 (NR)	NR	–34.9	–0.2	.17	0
Total direct obstetric causes (A34, O00-O92)	1109 (13.2)	12.4-13.9	458 (16.8)	15.3-18.4	27.7	3.7	<.001	60 (13.1)
Pregnancy with abortive outcome (O00-O07)	61 (0.7)	0.5-0.9	21 (0.8)	0.4-1.1	6.5	0.1	.80	1 (4.8)
Hypertensive disorders (O10-O16)	158 (1.9)	1.6-2.2	71 (2.6)	2.0-3.2	39.0	0.7	.02	7 (9.9)
Obstetric hemorrhage (O20, O43.2, O44-O46, O67, O71.0, O71.1, O71.3, O71.4, O71.7, O72)	92 (1.1)	0.9-1.3	38 (1.4)	1.0-1.8	27.8	0.3	.21	0
Pregnancy-related infection (O23, O41.1, O75.3, O85, O86, O91)	28 (0.3)	0.2-0.5	12 (NR)	NR	32.6	0.1	.41	0
Venous thrombosis in pregnancy (O22.0, O22.2, O22.3, O22.5, O22.9)	26 (0.3)	0.2-0.4	8 (NR)	NR	–4.8	–0.0	.90	1 (12.5)
Diabetes in pregnancy (O24)	30 (0.4)	0.2-0.5	19 (0.7)	0.4-1.0	95.9	0.3	.02	4 (21.1)
Liver disorders in pregnancy (O26.6)	66 (0.8)	0.6-1.0	25 (0.9)	0.6-1.3	17.2	0.1	.50	1 (4.0)
Other specified pregnancy-related conditions (O26.8)	397 (4.7)	4.3-5.2	190 (7.0)	6.0-8.0	48.0	2.3	<.001	43 (22.6)
Other complications of obstetric surgery and procedures (O75.4)	40 (0.5)	0.3-0.6	7 (NR)	NR	–45.9	–0.2	.13	1 (14.3)
Obstetric embolism (O88)	86 (1.0)	0.8-1.2	27 (1.0)	0.6-1.3	–2.9	–0.0	.77	0
Cardiomyopathy in the puerperium (O90.3)	61 (0.7)	0.5-0.9	22 (0.8)	0.5-1.1	11.6	0.1	.66	0
Total indirect causes (O98-O99)	422 (5.0)	4.5-5.5	214 (7.9)	6.8-8.9	56.9	2.9	<.001	42 (19.6)
Other viral diseases (O98.5)	2 (NR)	NR	16 (0.6)	0.3-0.9	2374.7	0.6	<.001	16 (100.0)
Other maternal infectious and parasitic diseases (O98.8)	21 (0.3)	0.1-0.4	13 (NR)	NR	91.5	0.2	.06	5 (38.5)
Mental disorders and diseases of the nervous system (O99.3)	25 (0.3)	0.2-0.4	10 (NR)	NR	23.7	0.1	.57	1 (10.0)
Diseases of the circulatory system (O99.4)	133 (1.6)	1.3-1.9	74 (2.7)	2.1-3.3	72.1	1.1	.001	1 (1.4)
Diseases of the respiratory system (O99.5)	27 (0.3)	0.2-0.4	19 (0.7)	0.4-1.1	117.7	0.4	.01	11 (57.9)
Other specified diseases and conditions (O99.8)	183 (2.2)	1.9-2.5	73 (2.7)	2.1-3.3	23.4	0.5	.13	6 (8.2)
Total nonspecific causes (O26.8, O95, O99.8)	637 (7.6)	7.0-8.2	275 (10.1)	8.9-11.3	33.5	2.5	.001	49 (17.8)
Total coded to late maternal causes after 42 d or within 1 y (O96)	691 (8.2)	7.6-8.8	315 (11.6)	10.3-12.8	41.0	3.4	<.001	34 (10.8)

Abbreviations: *ICD-10*, *International Statistical Classification of Diseases and Related Health Problems, Tenth Revision*; NR, not reliable.

^a^ Per 100 000 live births in 2018, 2019, and January to March (quarter 1) 2020.

^b^ Per 100 000 live births in April to December (quarters 2-4) 2020.

^c^ *ICD-10* codes generally followed World Health Organization *ICD–Maternal Mortality* categories, with hypertensive, diabetes, or venous complications grouped according to their ICD chapter or specific cause code. Residual categories are not shown to save space and promote clarity of presentation.

^d^ Any rates with a numerator fewer than 16 deaths were suppressed because of limited reliability determined by SEs that would be greater than 25% of the rate itself under the assumption of a Poisson process. Relative change and absolute change were reported when at least 1 period met the reliability standards.

^e^ Calculated as follows: ([rate during pandemic] − [rate prepandemic])/(rate prepandemic).

^f^ Based on *z* test of proportions.

^g^ Per maternal deaths for a given cause in April to December 2020.

**Figure.**
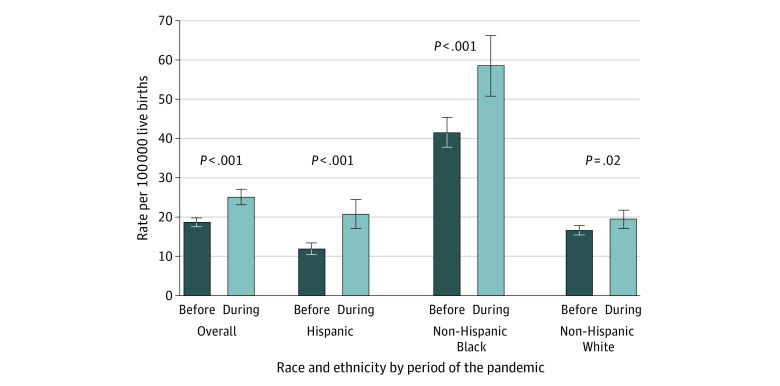
US Maternal Mortality Rates (95% CI) Before and During the COVID-19 Pandemic by Race and Ethnicity All rates met standards of reliability (numerator >16 deaths), which corresponded to SEs that were below 25% of the rate under the assumption of a Poisson distribution. *P* values were based on *z* test of proportions comparing the period during the pandemic (April-December 2020) with the period before the pandemic (2018, 2019, and January-March 2020).

For underlying cause-of-death codes (Table), the largest relative increase was among indirect causes (56.9%), specifically other viral diseases (2374.7%), diseases of the respiratory system (117.7%), and diseases of the circulatory system (72.1%). Relative increases in direct causes (27.7%) were mostly associated with diabetes in pregnancy (95.9%), hypertensive disorders (39.0%), and other specified pregnancy-related conditions (48.0%). COVID-19 was commonly listed as a secondary condition with other viral diseases (16 of 16 deaths [100%]) and diseases of the respiratory system (11 of 19 deaths [57.9%]) (Table). Almost half of those with a secondary code for COVID-19 (49 of 102) had a nonspecific code (*ICD-10* O26.8 or O99.8) as the underlying cause.

## Discussion

In the US, maternal deaths increased substantially (33.3%) after March 2020, corresponding to COVID-19 onset, a figure higher than the 22% overall excess death estimate associated with the pandemic.^4^ Increases were highest for Hispanic and non-Hispanic Black women. Change in maternal deaths during the pandemic may involve conditions directly related to COVID-19 (respiratory or viral infection) or conditions exacerbated by COVID-19 or other health care disruptions (diabetes or cardiovascular disease)^5^ but could not be discerned from the data.

Study limitations include the large percentage of COVID-19 cases with a nonspecific underlying cause (reflecting a maternal death coding problem^6^) and partitioning of data that resulted in small numbers for some categories (rates were suppressed for <16 deaths). Future studies of maternal death should examine the contribution of the pandemic to racial and ethnic disparities and should identify specific causes of maternal deaths overall and associated with COVID-19.

## Notes

Supplement.
eMethods.

## References

[1] Hoyert DL. Maternal mortality rates in the United States, 2020. Health E-Stat. February 23, 2022. Accessed May 5, 2022. https://www.cdc.gov/nchs/data/hestat/maternal-mortality/2020/E-stat-Maternal-Mortality-Rates-2022.pdf

[2] Murphy SL, Kochanek KD, Xu J, Arias E. Mortality in the United States, 2020. NCHS Data Brief. 2021;(427):1-8. 34978528

[3] Hoyert DL, Miniño AM. Maternal mortality in the United States: changes in coding, publication, and data release, 2018. Natl Vital Stat Rep. 2020;69(2):1-18. 32510319

[4] Woolf SH, Chapman DA, Sabo RT, Zimmerman EB. Excess deaths from COVID-19 and other causes in the US, March 1, 2020, to January 2, 2021. JAMA. 2021;325(17):1786. doi:10.1001/jama.2021.5199 33797550 PMC8019132

[5] Pérez-López FR, Savirón-Cornudella R, Chedraui P, . Obstetric and perinatal outcomes of pregnancies with COVID 19: a systematic review and meta-analysis. J Matern Fetal Neonatal Med. Published online March 13, 2022. doi:10.1080/14767058.2022.2051008 35282784

[6] MacDorman MF, Thoma M, Declercq E. Improving US maternal mortality reporting by analyzing literal text on death certificates, United States, 2016-2017. PLoS One. 2020;15(10):e0240701. doi:10.1371/journal.pone.0240701 33112910 PMC7592741

