# Supplementary material for: All-Cause Maternal Mortality in the US Before vs During the COVID-19 Pandemic
Source: JAMA Netw Open. 2022 Jun 28;5(6):e2219133. doi: 10.1001/jamanetworkopen.2022.19133 (PMC9240902; doi:10.1001/jamanetworkopen.2022.19133)
Supplement: Supplement. — eMethods. [file jamanetwopen-e2219133-s001.pdf]

Supplementary Online Content

Thoma ME, Declercq ER. All-cause maternal mortality in the US before vs during the COVID-19 pandemic. *JAMA Netw Open*. 2022;5(5):e2219133.
doi:10.1001/jamanetworkopen.2022.19133

eMethods.

This supplementary material has been provided by the authors to give readers additional information about their work.

© 2022 Thoma ME et al. *JAMA Network Open.*

eMethods

Definitions

Maternal mortality is defined by the World Health Organization (WHO) as "The death of a woman while pregnant or within 42 days of the termination of pregnancy, irrespective of the duration and site of the pregnancy, from any cause related to or aggravated by the pregnancy or its management but not from accidental or incidental causes."[1] A late maternal death is defined as "The death of a woman from direct or indirect causes more than 42 days but less than one year after termination of pregnancy." Maternal deaths are further subdivided into direct and indirect obstetric deaths. Direct obstetric deaths are "those resulting from obstetric complications of the pregnancy state (pregnancy, labor, and the puerperium), from interventions, omissions, incorrect treatment, or from a chain of events resulting from any of the above." Indirect obstetric deaths are "those resulting from previous existing disease or disease that developed during pregnancy and which was not due to direct obstetric causes, but which was aggravated by the physiologic effects of pregnancy."

ICD-10 groups

To facilitate comparisons of maternal mortality statistics generated from ICD-10 data, WHO developed a grouping system for identification of maternal deaths using existing ICD-10 codes (ICD-MM).[1] ICD-MM groups include: Pregnancies with abortive outcome (Group 1), Hypertensive disorders in pregnancy, childbirth, and puerperium (Group 2), Obstetric hemorrhage (Group 3), Pregnancy related infection (Group 4), Other obstetric complications (Group 5), Unanticipated complications of management (Group 6), Non-obstetric complications (Group 7), and Unknown/undetermined (Group 8). Groups 1-6 are considered direct, Group 7 are indirect, and Group 8 is unknown/undetermined maternal deaths.

Classifying deaths of COVID-19 complicating pregnancy or the puerperium

Although pregnancy or puerperium complicated by COVID-19 is considered an indirect maternal death as an underlying cause by the World Health Organization,[2] the underlying cause would not be classified as COVID-19 (U07.1). Instead, guidelines state that these deaths should be classified as O98.5 (other viral diseases) or O99.5 (diseases of the respiratory system) with U07.1 additionally documented as a multiple cause. Alternatively, COVID-19 (U07.1) may also be listed in the multiple cause section if it was a contributory cause (i.e., predisposed an individual to death, but did not initiate the chain of events leading to the death).

Study Methods

In this study, we apply standard maternal mortality definitions using coding procedures established by the National Center for Health Statistics (NCHS) to classify maternal deaths in the U.S. for years 2018 onward (also referred to as the 2018 method).[3] For cause-specific mortality, we present groupings of ICD-10 codes for maternal deaths that generally correspond to ICD-MM. Given the indistinctness of ICD-MM Group 5 (Other obstetric conditions) and ICD-MM Group 7 (non-obstetric complications), we present the most common ICD-10 codes within that group and present all hypertensive disorders under its specific ICD-10 Chapter and diabetes or venous complications by their relevant ICD-10 codes, consistent with prior studies.[4] Residual categories are not shown in Table 1 to save space and promote clarity of presentation.

© 2022 Thoma ME et al. *JAMA Network Open.*

Consistent with prior studies examining excess deaths from COVID-19,[5] we examine multiple cause data to capture the universe of COVID-19 involved maternal deaths. The time periods of comparison include before the COVID-19 pandemic (2018, 2019, and January-March of 2020) and during the pandemic (April-December of 2020). Prior to collapsing across the pre-pandemic time periods, we compared overall and race/ethnic-specific maternal mortality rates across different quarters (Jan-March, April-December) and years (2018-2020) and found no consistent pre-pandemic trend or pattern in maternal mortality rates, thus we collapsed over these time periods. Any rates with numerators lower than 16 were suppressed (corresponding to a standard error for the rate that would be 25% or larger than the rate itself), consistent with other suppression criteria for rates due to concerns over reliability (https://www.cdc.gov/cancer/uscs/technical_notes/stat_methods/suppression.htm).

eReferences

1. World Health Organization. Regional Office for Africa. *The WHO Application of ICD-10 to Deaths during Pregnancy, Childbirth and the Puerperium: ICD-MM*. World Health Organization; 2012:78. Accessed May 5, 2022. https://apps.who.int/iris/bitstream/handle/10665/70929/9789241548458_eng.pdf

2. World Health Organization. *Medical Certification, ICD Mortality Coding, and Reporting Mortality Associated with COVID-19*.; 2020. Accessed May 5, 2022. https://www.who.int/publications-detail-redirect/WHO-2019-nCoV-mortality-reporting-2020-1

3. Hoyert D, Minino A. *Maternal Mortality in the United States: Changes in Coding, Publication, and Data Release, 2018*. National Center for Health Statistics; 2020:18.

4. MacDorman MF, Thoma M, Declercq E. Improving US maternal mortality reporting by analyzing literal text on death certificates, United States, 2016-2017. *PloS One*. 2020;15(10):e0240701. doi:10.1371/journal.pone.0240701

5. Woolf SH, Chapman DA, Sabo RT, Zimmerman EB. Excess Deaths From COVID-19 and Other Causes in the US, March 1, 2020, to January 2, 2021. *JAMA*. 2021;325(17):1786. doi:10.1001/jama.2021.5199

© 2022 Thoma ME et al. *JAMA Network Open.*
